# Serum bta-miRNA-375 as a potential biomarker for the early diagnosis of enzootic bovine leukosis

**DOI:** 10.1371/journal.pone.0302868

**Published:** 2024-05-09

**Authors:** Kenji Murakami, Towa Matsunaga, Takashi Matsuzaki, Yuta Naruke, Sonoko Miyauchi, Sota Kobayashi, Syuji Yoneyama, Yusuke Sakai, Toshihiro Ichijo, Toh-ichi Hirata, Atsushi Kimura, Yuzumi Chiba, Kei-ich Matsuda, Shinji Yamada, Hirokazu Hikono

**Affiliations:** 1 Graduate School of Veterinary Sciences, Iwate University, Morioka, Iwate, Japan; 2 Faculty of Agriculture, Farm Animal Clinical Skill and Disease Control Center, Iwate University, Morioka, Iwate, Japan; 3 National Veterinary Assay Laboratory, Ministry of Agriculture, Forestry and Fisheries, Kokubunji, Tokyo, Japan; 4 Food Safety and Consumer Affairs Bureau, Ministry of Agriculture, Forestry and Fisheries, Chiyoda, Tokyo, Japan; 5 Animal Diagnostic Laboratory, Ehime Prefecture, Toon, Ehime, Japan; 6 National Institute of Animal Health, Tsukuba, Ibaraki, Japan; 7 Faculty of Agriculture, Field Science Center, Iwate University, Shizukuishi, Iwate, Japan; 8 Iwate Central Livestock Hygiene Center, Morioka, Iwate, Japan; 9 Livestock Medicine Training Center, Miyagi Prefecture Agricultural Mutual Aid Association, Oohira, Miyagi, Japan; 10 Faculty of Life and Environmental Sciences, Department of Animal Sciences, Teikyo University of Science, Adachi, Tokyo, Japan; University of Verona, ITALY

## Abstract

To identify a biomarker for the early diagnosis of enzootic bovine leukosis (EBL) caused by bovine leukemia virus (BLV), we investigated the expression of a microRNA, bta-miR-375, in cattle serum. Using quantitative reverse-transcriptase PCR analysis, we measured bta-miR-375 levels in 27 samples from cattle with EBL (EBL cattle), 45 samples from animals infected with BLV but showing no clinical signs (NS cattle), and 30 samples from cattle uninfected with BLV (BLV negative cattle). In this study, we also compared the kinetics of bta-miR-375 with those of the conventional biomarkers of proviral load (PVL), lactate dehydrogenase (LDH), and thymidine kinase (TK) from the no-clinical-sign phase until EBL onset in three BLV-infected Japanese black (JB) cattle. Bta-miR-375 expression was higher in NS cattle than in BLV negative cattle (*P* < 0.05) and greater in EBL cattle than in BLV negative and NS cattle (*P* < 0.0001 for both comparisons). Receiver operating characteristic curves demonstrated that bta-miR-375 levels distinguished EBL cattle from NS cattle with high sensitivity and specificity. In NS cattle, bta-miR-375 expression was increased as early as at 2 months before EBL onset—earlier than the expression of PVL, TK, or LDH isoenzymes 2 and 3. These results suggest that serum miR-375 is a promising biomarker for the early diagnosis of EBL.

## Introduction

The several causes of lymphoma in cattle include the retrovirus bovine leukemia virus (BLV), which causes enzootic bovine leukosis (EBL) [1]. EBL is a malignant B-cell lymphoma in cattle caused by BLV; this RNA virus belongs to the genus *Deltaretrovirus*, family *Retroviridae*, and is closely related to human T-lymphotropic virus types 1 and 2 [1,2]. The majority of BLV-infected cattle show no clinical signs. Approximately 30% of BLV-infected cattle develop persistent lymphocytosis, and 0.1% to 5% of these develop EBL [1,3]. EBL is reportable to the World Organization for Animal Health, with disease incursion affecting international trade. In Japan, EBL is designated as a reportable disease according to the Act on Domestic Animal Infectious Diseases Control (https://www.japaneselawtranslation.go.jp/en/laws/view/4114, accessed on December 4, 2023).

Diagnosing EBL before cattle show characteristic clinical signs is difficult, because no obvious clinical signs occur during the long subclinical infection period [1]. On-farm diagnoses typically involve the documentation of enlarged superficial lymph nodes, protrusion of the eye, palpation of a tumor mass on rectal examination, increased peripheral blood lymphocytes, and confirmation of neoplastic B cells in blood smears. More than half of EBL cattle are detected by postmortem inspections at slaughterhouses, not through on-farm inspections [4]. EBL cattle cannot be distributed for human consumption, so the entire carcass is destroyed, thus causing great economic loss to farmers. Therefore, novel biomarkers are crucial for the early diagnosis of EBL, i.e., before cattle show classic clinical signs. Using such biomarkers during on-farm inspections will facilitate early culling of infected animals, thereby decreasing the incidence of EBL within the herd and the related economic loss.

Several biomarkers are associated with the onset of EBL. Serum levels of total lactate dehydrogenase (LDH) and its isozymes, LDH2 and LDH3, were higher in EBL cattle than in non-EBL cattle [5]. Thymidine kinase (TK) activity was higher in cattle with EBL for which clinical diagnosis was confirmed [6]. However, although serum levels of LDH and TK are associated with EBL onset, these markers are not increased in all EBL cattle [5,6]. Proviral load (PVL) in the lymph nodes or peripheral blood were higher in cattle with EBL than in animals that were BLV-infected but showed no clinical signs (i.e., NS cattle) [7,8]. Konishi et al. simultaneously evaluated LDH, TK, and PVL levels in blood samples from cattle at various clinical stages of BLV infection—i.e., EBL, persistent lymphocytosis, NS, and BLV negative—and suggested that the LDH2 isozyme and TK were potential biomarkers for the diagnosis of EBL [9]. However, how early these biomarkers are elevated before EBL onset has not previously been investigated.

MicroRNAs (miRNAs) are endogenous single-stranded non-coding RNAs that selectively bind to complementary sites in the 3′ untranslated region of mRNAs and influence their cleavage and translation [10]. Many studies have demonstrated that miRNAs are involved in the pathogenesis of cancer [11] as so-called onco-miRNAs or tumor-suppressor miRNAs [12]. In addition, miRNAs have been detected in biofluids such as serum and have attracted attention as novel biomarkers for cancer diagnosis and prognosis [13–16]. In our previous study, we demonstrated that most host-derived miRNAs (bta-miRNAs) were significantly decreased in BLV-infected cattle compared with BLV-uninfected (BLV negative) cattle, but only bta-miR-375 was significantly increased in the B cells and B-cell lymphoma tissues of BLV-infected cattle [17]. In addition, bta-miR-375 was significantly increased in the white blood cells (WBC) of BLV-infected compared with BLV negative cattle [18].

In the present study, we investigated serum bta-miR-375 as a novel biomarker for the early diagnosis of EBL. First, we compared the expression of bta-miR-375 in serum among cattle with EBL, those infected with BLV but lacking clinical signs (NS cattle), and those uninfected with BLV (BLV negative cattle). Second, we compared the kinetics of bta-miR-375 with those of PVL, TK, and LDH2 in BLV-infected cattle to determine the timing of increased bta-miR-375 expression from the preclinical phase through EBL onset.

## Materials and methods

### Animals

We evaluated 109 bovine blood and lymphoma tissue samples comprising 28 samples from cattle with EBL (14 Japanese Black [JB], 13 Holstein–Friesian [HF], and 1 JB × HF crossbred), 49 from NS cattle (24 JB and 25 HF), and 32 from BLV negative cattle (18 JB, 10 HF, and 4 Jersey cattle) (S1 Table). These samples were collected at cattle breeding farms in Ehime, Hokkaido, Iwate, and Miyagi prefectures from May 2017 through June 2021. In addition, 3 JB cattle (J04, J19, and J24) with BLV PVL of more than 400 copies per 10 ng DNA and that were born and raised at the Iwate University Field Science Center were used for the kinetics analysis of bta-miR-375 expression. BLV negative cattle were defined as those negative for BLV provirus according to quantitative polymerase chain reaction (qPCR) analysis and for antibodies to BLV according to enzyme-linked immunosorbent assay (ELISA).

EBL onset was confirmed through postmortem autopsy at the Department of Veterinary Medicine, Faculty of Agriculture, Iwate University. Autopsied cattle had numerous enlarged lymph nodes, including iliac, mesenteric, and superficial lymph nodes, and masses in the abdominal cavity. The cut surfaces of these lymph nodes and masses were homogeneously milky white, pulp-like, bulging, and partially necrotic. Most cells found in lymphoma tissue were cytoplasm-poor, round cells with round nuclei containing aggregated chromatin and CD3-negative, CD20-positive B cells. In addition, Giemsa staining of peripheral blood smears showed that more than 10% of lymphocytes were atypical, with nuclei that were constricted or flower-like in morphology; nuclei were moderately sized, with a diameter equivalent to two or three erythrocytes.

In addition, we confirmed the presence of BLV provirus and anti-BLV antibodies in autopsied cattle by using qPCR and ELISA, and blood leukocyte counts were determined by using an automated blood cell analyzer (MEK-6558, Nihon Kohden, Tokyo, Japan).

All procedures involving animals were approved by the Iwate University Animal Care and Use Committee (no. A201704) and were performed in accordance with the Iwate University Animal Experimentation Regulations. All methods in this study are reported in accordance with ARRIVE guidelines (https://arriveguidelines.org/arrive-guidelines, accessed on December 4, 2023).

### Histopathology and immunohistochemistry

Lymphoma tissues were fixed by 10% neutral-buffered formaldehyde. Paraffin sections (4 μm thickness) were deparaffinized and hydrated with xylene and graded alcohol solutions. Paraffin sections were stained with hematoxylin and eosin (HE). For immunohistochemistry, antigen retrieval was performed by immersing the sections in 10 mM citrate buffer (pH 6.0) in a pressure cooker and autoclaving for 20 min and then cooled to room temperature. For removal of endogenous peroxidase, sections are immersed in 0.3% hydrogen peroxide/methanol solution and incubated at room temperature for 30 minutes and then incubated with an optimal diluted primary antibody (rabbit polyclonal anti-human CD3 for T cell (M7254, Dako Japan, Tokyo, Japan) or CD20 for B cell (RB-9013-P1, Thermo Fisher Scientific JP, Tokyo, Japan) for 30 min at room temperature. Secondary staining was achieved with a Dako LSAB+ kit (Dako Japan) and developed with 3, 39-diaminobenzidine tetrahydrochloride and chromogen (Liquid DAB+ Substrate Chromogen System, K3468, Dako Japan) according to manufacturer’s instruction. Sections were counterstained for 1 min with hematoxylin and then mounted.

### Quantification of BLV provirus and BLV antibody

Genomic DNA was extracted from EDTA-containing blood samples by using a fully automated nucleic acid extractor (magLEAD 12GC, Precision Science Systems, Chiba, Japan). DNA concentration was calculated according to absorbance at 260 nm (Nano Drop One, Thermo Fisher Scientific, Waltham, MA, USA).

Duplex qPCR analysis targeting the BLV *tax* gene and bovine *RNase P* gene (internal control) was used to measure BLV PVL, as previously described [17]. PVL is indicated as the number of BLV copies per 10 ng DNA.

Anti-BLV antibodies in blood samples were measured by using ELISA (Nippon Gene, Toyama, Japan) according to the manufacturer’s instructions.

### Extraction of total RNA from serum, B cells, and lymphoma tissue

Peripheral blood was collected from the jugular vein twice each month. Serum was separated by centrifuging clotted blood at 1080 × *g* for 20 min at 4°C and stored at −20°C. Serum was thawed and centrifuged at 16,000 × g for 10 min at 4°C, and the supernatant was collected to remove nucleotides bound to cell debris. miRNAs were extracted from the collected serum by using an miRNeasy Serum/Plasma Advanced kit (Qiagen KK, Tokyo, Japan) according to the manufacturer’s protocol. We added 5.6 × 10^8^ copies of synthetic exogenous *Caenorhabditis elegans* miR-39 (cel-miR-39-3p; Qiagen) to samples to facilitate evaluation of miRNA extraction efficiency and data normalization. RNA extraction from B cells and lymphoma tissues was performed as previously reported [17]. Extracted RNA was evaluated at absorbance ratios of 260/280 nm and 260/230 nm (Nano-Drop One, Thermo Fisher Scientific).

### Quantification of bta-miR-375

cDNA was obtained by reverse transcription of total RNA (miScript II RT kit, Qiagen). The reaction solution comprised 2 μL of 5× miScript HiFlex buffer, 1 μL of 10× miScript Nucleics Mix, 1 μL of miScript Reverse Transcriptase Mix, 5 μL of total RNA, and RNase-free water to yield a total volume of 10 μL. The obtained cDNA was diluted 200-fold with RNase-free water and used for qPCR analysis.

qPCR analysis was performed by using miScript SYBR Green PCR kit (Qiagen). The forward primers were: bta-miR-375, 5′-TTTTGTTCGTTCGGCTCG-3′, and cel-miR-39-3p, 5′-TCACCGGGTGTAAATCAGCTTG-3′. We used the universal primer supplied with the kit as the reverse primer. The reaction mixture contained 12.5 μL of 2× Quantitect SYBR Green PCR master mix, 2.5 μL of 10× miScript universal primer, 2.5 μL of each forward primer (5 μM), 6.5 μL of sterile ultrapure water, and 1.0 μL of the diluted cDNA solution. The conditions for real-time PCR analysis (QuantStudio 3, Thermo Fisher Scientific) were initial denaturation at 95°C for 15 min, followed by thermal denaturation at 94°C for 15 s, annealing at 55°C for 30 s, and elongation at 70°C for 30 s for a total of 40 cycles. To assess for non-specific amplification products, we performed a melting curve analysis (QuantStudio 3 software, version 1.7, Applied Biosystems, Thermo Fisher Scientific). All samples were tested in duplicate and were normalized to the synthetic spike-in control, cel-miR-39-3p (Qiagen). Relative miRNA expression was calculated by using Gene Expression suite software (version 1.3, Thermo Fisher Scientific).

### TK activity

We used a commercially available ELISA kit (DiviTum V2, BIOVICA International, Uppsala, Sweden) according to the manufacturer’s instructions to measure TK activity in serum samples. TK activity was determined relative to the activity of a reference sample of recombinant TK in serum and expressed as DiviTum Units per liter (Du/L); as reported by the manufacturer, 1000 Du/L corresponds to the activity obtained from 1000 ng TK/L.

### LDH isozymes

Total LDH activity in serum samples was measured according to the two-wavelength rate method by using an automated biochemical analyzer (Dimension RL Max, Siemens Healthcare, Tokyo, Japan). Serum LDH isozymes were measured via agarose gel electrophoresis by using an automated agarose electrophoresis analyzer (Epalyzer 2, Helena Laboratories Japan, Saitama, Japan). These were measured at the NOSAI Miyagi Livestock Medicine Training Center (Oohira, Miyagi, Japan).

### Statistical analysis

Relative quantities of serum bta-miR-375 in EBL, NS, and BLV negative cattle were compared by using the Kruskal–Wallis test and Dunn’s multiple-comparison test. Relative quantities of serum bta-miR-375 were compared between JB EBL cattle and HF EBL cattle by using the Mann–Whiteny U test. Correlation between bta-miR-375 concentration and PVL was assessed according to Spearman’s correlation coefficient by using a rank test. Receiver operating characteristic (ROC) analysis using 45 NS and 27 EBL cattle was performed to assess the discriminative capacity of bta-miR-375 in identifying EBL. When miR-375 was undetectable in serum, the data were indicated as a value of 0 for purposes of comparison. The kinetics of miR-375, PVL, peripheral blood leukocyte counts, TK, LDH2, LDH3, and LDH2+3 from the no-clinical-sign phase until the onset of EBL in three JB cattle were compared by using the Friedman test and Dunn multiple-comparison test. All statistical analyses were performed by using Prism 9 (GraphPad Software, La Jolla, CA, USA), and *P* < 0.05 was considered to indicate a significant difference.

## Results

### Comparison of serum expression of bta-miR-375 in EBL, NS, and BLV negative cattle

**Fig 1:**
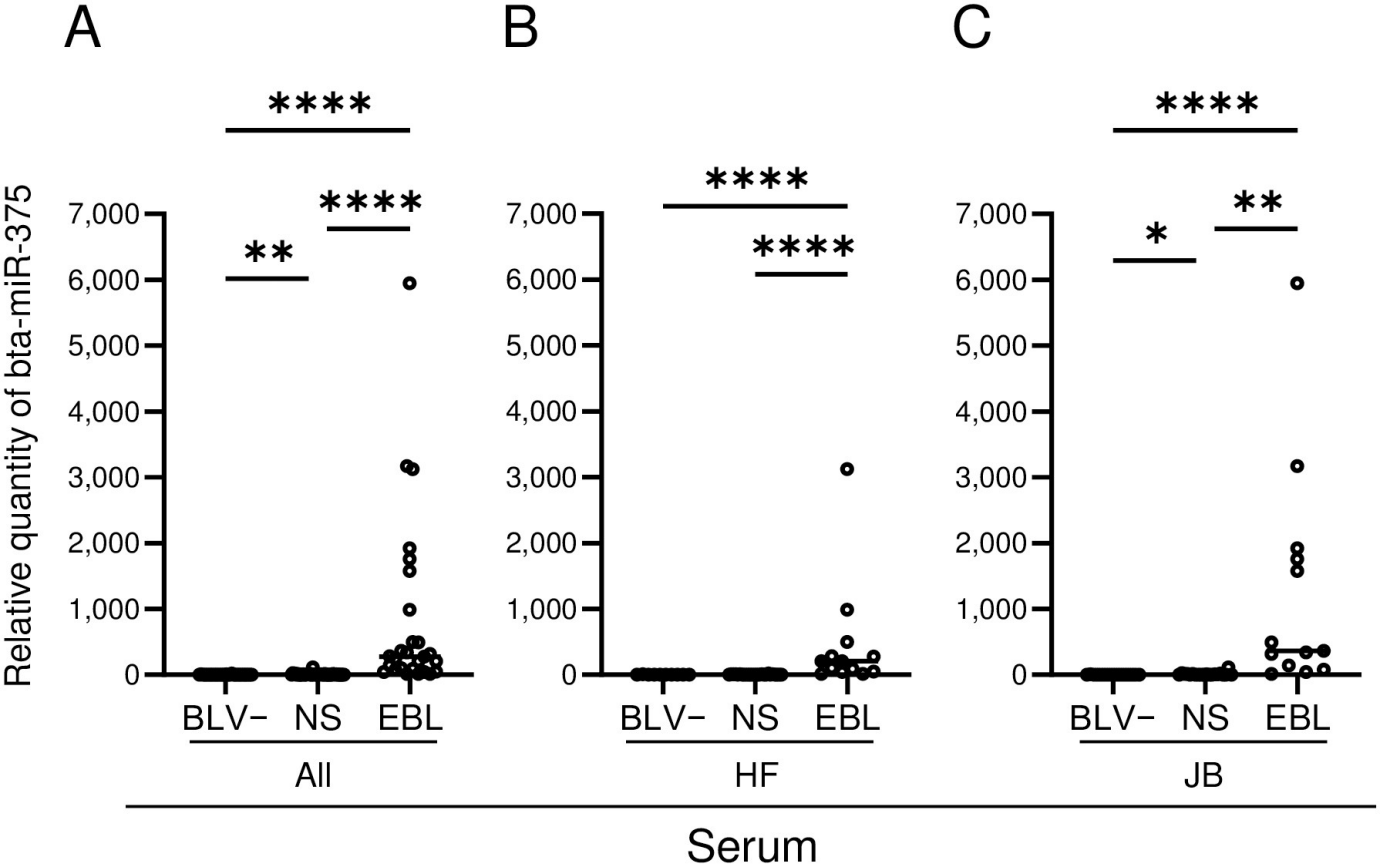
bta-miR-375 in sera and tissues of BLV-infected and -negative cattle according to quantitative reverse transcriptase–PCR analysis. A: All cattle (n = 102), B: Holstein–Friesian (HF) cattle (n = 48), C: Japanese Black (JB) cattle (n = 49). Serum data are indicated as relative quantities normalized to the synthetic spike-in control, cel-miR-39-3p. The values indicated as ‘undetectable’ were defined as 0 for the purposes of comparison. BLV−, bovine leukemia virus (BLV) negative cattle; NS, cattle infected with BLV but showing no clinical signs; EBL, cattle with enzootic bovine leukosis (EBL). *: *P* < 0.05, **: *P* < 0.001, ***: *P* < 0.001, ****: *P* < 0.0001. Horizontal bars indicate median values.

The expression of bta-miR-375 in serum (Fig 1) was significantly higher in NS cattle than in BLV negative cattle (*P* < 0.05) and significantly greater in EBL cattle than in either BLV negative or NS cattle (*P* < 0.0001 for both comparisons). This same trend in expression among the three groups was maintained in JB cattle, but in HF cattle, bta-miR-375 expression levels were similar between NS and BLV negative cattle. No significant differences were observed in the expression of bta-miR-375 between JB EBL cattle and HF EBL animals.

**Fig 2:**
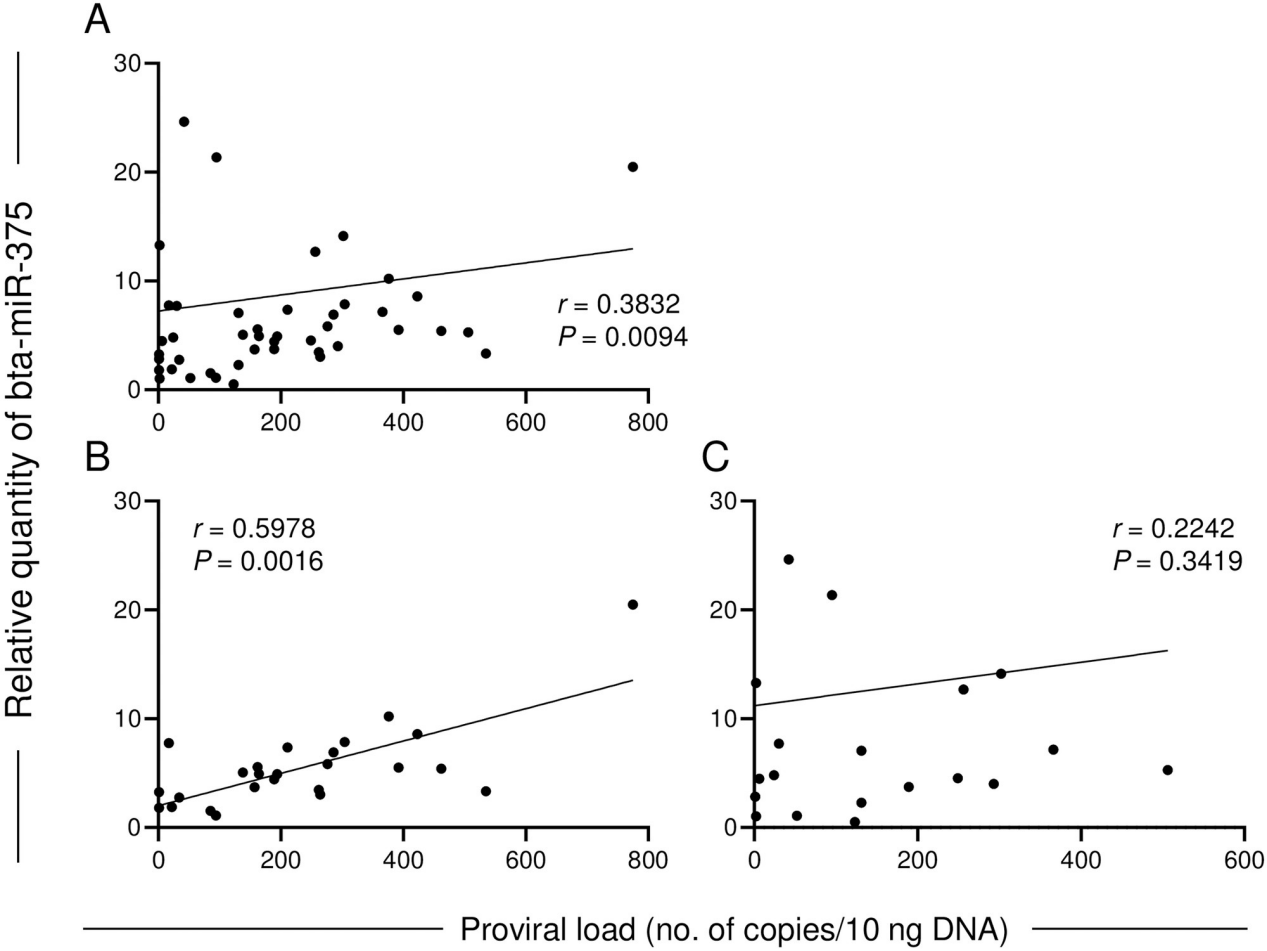
Correlation between bta-miR-375 and proviral load (PVL) in cattle infected with BLV but without clinical signs. A: All cattle, B: HF cattle, C: JB cattle. bta-miR-375 levels are indicated as relative quantities normalized to the synthetic spike-in control, cel-miR-39-3p. PVL is indicated as the number of BLV copies per 10 ng DNA. Data were analyzed by using Spearman’s correlation coefficient test.

Overall, bta-miR-375 expression was weakly correlated with PVL (*r* = 0.3586, *P* = 0.0156; Fig 2), with moderate correlation between bta-miR-375 and PVL in HF cattle (*r* = 0.5692, *P* = 0.0030) but no correlation between these factors in JB cattle (*r* = 0.2126, *P* = 0.3683). No correlation between miR-375 expression and age was observed (S1 Fig).

### Correlation between bta-miR-375 expression in serum and B cell or lymphoma tissue from EBL, NS, and BLV negative cattle

Bta-miR-375 expression in serum was strongly correlated with that in B cells or lymphoma tissue (*r* = 0.7818, *P* = 0.0105; S2 Fig).

### ROC analysis

**Fig 3:**
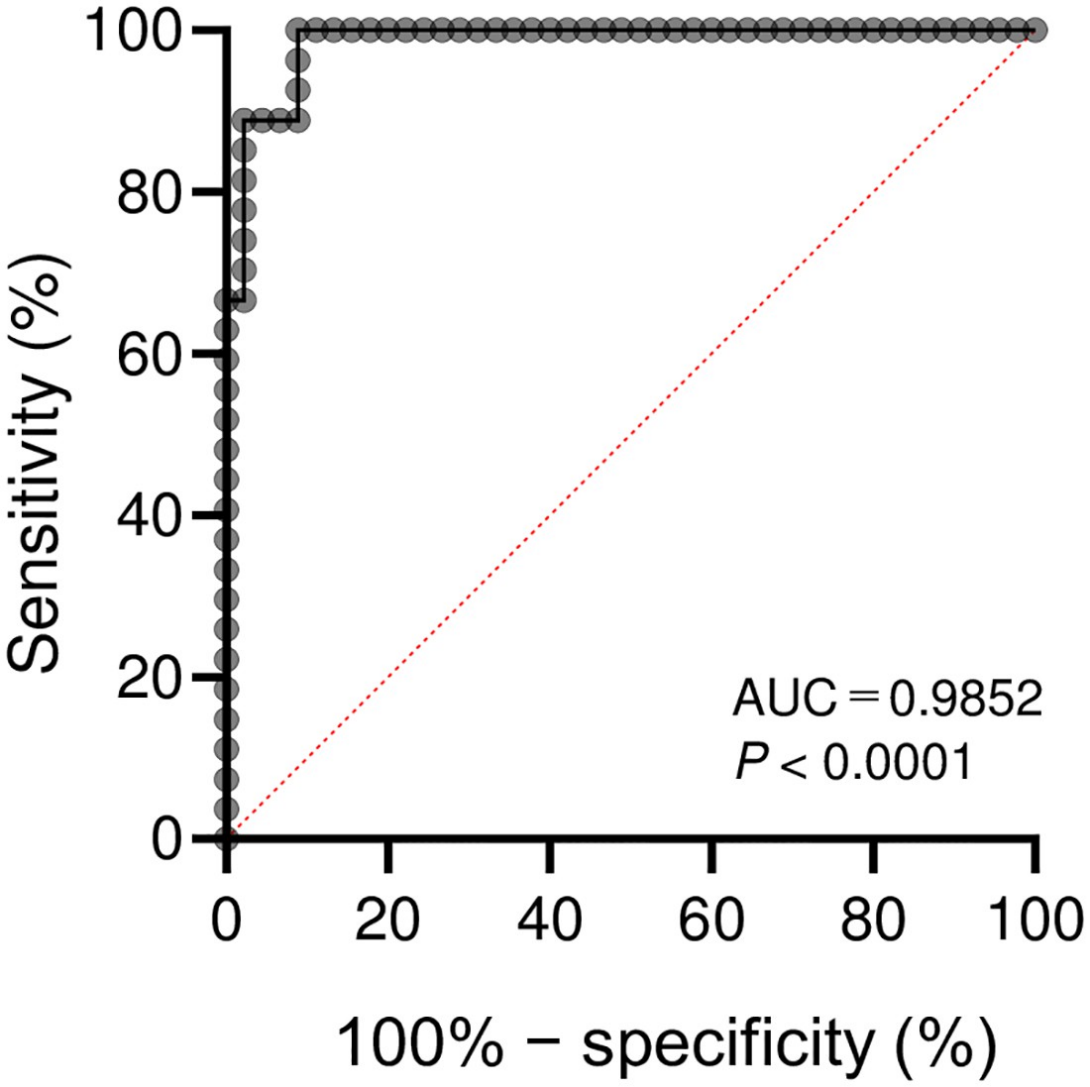
Receiver operating characteristic curve analysis of serum bta-miR-375 for distinguishing EBL cattle from NS cattle. AUC, area under the curve.

ROC analysis of the diagnostic performance of serum bta-miR-375 for EBL diagnosis yielded an area under the curve of 0.9852 (95% confidence interval, 0.9650–1.000) (Fig 3). A cutoff value of 14.5 gave a sensitivity of 100% and specificity of 91.11%.

### Kinetics of bta-miR-375, PVL, leukocyte count, TK, and LDH in BLV-infected cattle during the preclinical phase through EBL onset

**Fig 4:**
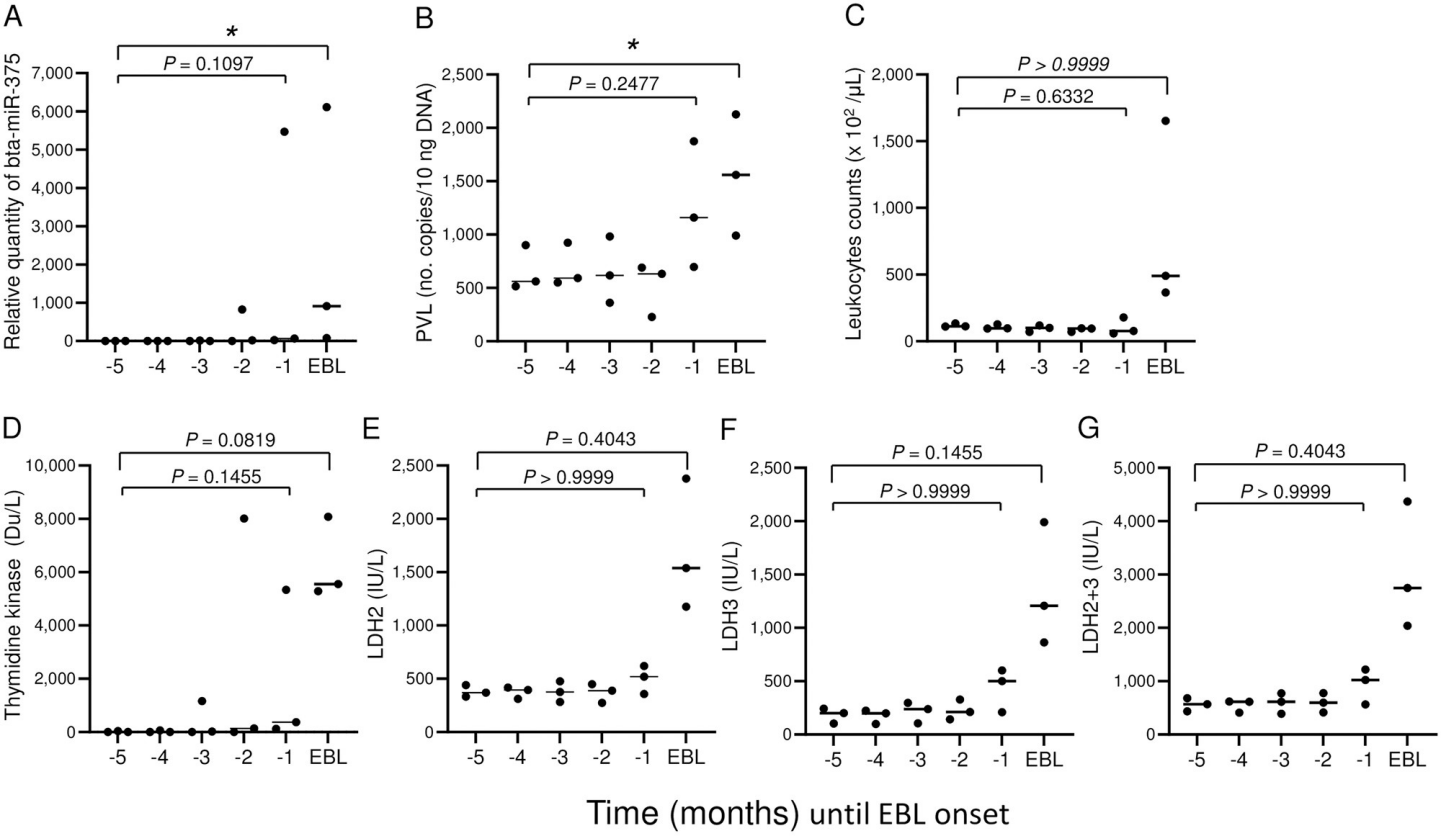
Kinetics of serum bta-miR-375, PVL, peripheral blood leukocytes, thymidine kinase (TK), and lactate dehydrogenase 2, 3, and 2+3 (LDH2, 3, and 2+3) levels from the preclinical phase to EBL onset in three BLV-infected cattle. A: bta-miR-375, B: PVL, C: Leukocyte count, D: TK, E: LDH2, F: LDH3, G: LDH2+3. bta-miR-375 and PVL levels were measured by using quantitative RT-PCR and quantitative PCR analysis, respectively; TK was measured by ELISA; and LDH was measured by using an automated biochemical analyzer. Horizontal bars indicate medians; *, *P* < 0.05.

Serum levels of bta-miR-375, PVL, peripheral blood leukocyte count, TK, LDH2, LDH3, and LDH2+3 tended to be higher at EBL onset than at 5 months before EBL onset (i.e., during the preclinical phase) (Fig 4). The expression of bta-miR-375 increased in 2 of the 3 BLV-infected cattle at 2 months before EBL onset and in all 3 animals at 1 month before EBL onset (Fig 4A). PVL was increased above the threshold level (1,000 copies/10 ng DNA) [7] in 2 of the 3 animals at 1 month before EBL onset (Fig 4B). Leukocyte counts were elevated only at onset of EBL (Fig 4C). TK was greater than the threshold level (100 Du/L) [9] in 1 of the 3 animals at 1, 2, and 3 months before EBL onset (Fig 4D). LDH2 and 3 exceeded the threshold level (500 IU/L) [9] in 2 of the 3 animals at 1 month before EBL onset (Fig 4E and 4F).

## Discussion

The results of the current study showed that serum bta-miR-375 was higher in EBL cattle than in NS or BLV negative cattle and greater in NS cattle than in BLV negative cattle. Bta-miR-375 expression in serum was strongly correlated with its expression in B cells or lymphoma tissue. In addition, ROC analyses demonstrated that bta-miR-375 levels distinguished EBL cattle from NS cattle with high sensitivity and specificity. Finally, the kinetics analysis of serum bta-miR-375 in BLV-infected cattle showed that serum bta-miR-375 was increased at 2 months before the onset of EBL.

The results of the ROC analysis suggest that serum bta-miR-375 can be used as a biomarker for the diagnosis of EBL. This conclusion is in harmony with previous reports in which miR-375 was a biomarker for the diagnosis and prognosis of leukemia in humans [19,20]. For example, miR-375 in peripheral blood was increased in patients with chronic myeloid leukemia [21] and in children with acute myeloid leukemia with poor prognosis [20].

Predicting which BLV-infected cattle will develop EBL and when they will do so is currently unfeasible. We here showed that NS cattle had greater levels of serum bta-miR-375 than BLV negative cattle. In addition, serum bta-miR-375 was increased at 2 months before EBL onset in 2 of the 3 BLV-infected cattle evaluated and in all 3 animals at 1 month before EBL onset. In contrast, conventional biomarkers for EBL diagnosis (e.g., PVL, leukocyte counts, TK, and LDH2 and 3), [9] were not consistently increased in BLV-infected cattle—even at 1 month before EBL onset. These results suggest that bta-miR-375 may be a predictive biomarker for the early diagnosis of EBL, i.e., before cattle show typical clinical signs.

In the current study, the level of bta-miR-375 was significantly higher in EBL cattle than in BLV-uninfected cattle. A recent study reported that the levels of bta-miR-17-5p, bta-miR-24-3p, and bta-miR-92a were significantly higher in HF EBL cattle than in BLV-uninfected cattle [22]. We cannot directly compare the results of these two studies because the methods to isolate RNA from serum samples differed. In particular, we isolated total RNA including miRNA directly from serum samples, whereas the other group isolated total RNA including miRNA from small extracellular vesicles (sEV) purified from serum samples. At least, these results imply that bta-miR-375 is not selectively encapsulated in serum sEV in HF EBL cattle.

A new method called RAISING-CLOVA has recently been proposed to be useful for the early prediction of EBL onset [23]. This method was used to analyze the clonality of BLV-infected cells (Cv) in 4 sheep with experimental BLV infection and showed that Cv increased before or at the EBL onset. In 3 of the 4 animals, Cv peaked earlier than PVL. It will be interesting to compare the Cv with bta-miR-375 and the conventional biomarkers such as PVL, TK, and LDH2 from the before clinical manifestations to EBL onset in BLV-infected cattle. Such studies will lead to the development of a new method for the early and precise prediction of EBL onset.

The role of miR-375 in the development of EBL is unknown, but several mRNA targets for miR-375 have been reported [24]. miR-375 was first discovered as a regulator of insulin secretion in pancreatic endocrine cells, [25] but its multiple regulatory functions are displayed in a broad variety of diseases in humans [24]. In particular, miR-375 may act as either a tumor suppressor or an oncogenic miRNA, depending on the type of tumor [24,26]. For example, miR-375 suppresses the growth and metastasis of colorectal cancer [27,28] and inhibits the proliferation and invasion of kidney cancer, melanoma, tongue squamous-cell carcinoma, and glioblastoma cells [29–33]. In contrast, miR-375 promotes tumorigenesis and progression in prostate cancer [34] and metastasis of small cell lung cancer [35]. Furthermore, dysregulation of miR-375 may reflect pathologic changes in tumors. For example, the pattern of miR-375 expression differs according to disease progression in patients with hepatocellular carcinoma [36], Kaposi’s sarcoma [37], metastatic medullary thyroid cancer [38], or pancreatic cancer [39]. Interestingly, we noted weak correlation between serum miR-375 and PVL levels in NS cattle. Previously, we reported that miR-375 expression in B cells increases with increasing PVL [17].

The mechanism of miR-375 upregulation in EBL is still unknown. Upregulation of miR-375 was reported to be a prerequisite for the induction of neuroendocrine features via the transcription factor achaete-scute complex homolog 1 (ASCL1), which is a basic-helix-loop-helix (bHLH) protein that is expressed in normal pulmonary neuroendocrine cells and in lung cancers with neuroendocrine features [40–42]. miR-375 was upregulated in and correlated with ASCL1 levels in small-cell lung cancer cells [42]. ASCL1 binds to three E-box elements (which are recognized by bHLH proteins) in the miR-375 promotor and thereby activates its expression [43,44]. Interestingly, the tumor suppressor genes p53 and YAP1 are direct targets of miR-375 [24], and miR-375 reduces the production of p53 and YAP1 [42,45]. In addition, several mutations in bovine p53 are associated with EBL onset [46,47]. Therefore, miR-375 may be upregulated by ASCL1, which itself may be stimulated by BLV infection through an as-yet-unknown mechanism. In addition, miR-375 may play a role in inhibiting tumor suppressors, such as p53 and YAP1, leading to lymphoma formation, or the level of miR-375 expression may reflect the disease stage in BLV-infected cattle. The molecular mechanism of bta-miR-375 regulation in EBL requires further investigation.

We acknowledge two limitations of the current study. We used only three animals for the kinetics analysis of bta-miR-375 in BLV-infected cattle. Continuously monitoring the expression of bta-miR-375 from the preclinical phase until the onset of EBL in a sufficient number of naturally BLV-infected cattle is difficult, because which and when BLV-infected cattle will develop EBL cannot be predicted. In fact, we needed to collect monthly blood samples from more than 100 BLV-infected cattle over several years to obtain the data from the three animals we reported in this study. To overcome this problem, a sheep model with experimental BLV infection might be useful, because BLV-infected sheep develop lymphoma at a higher frequency and more rapidly than do BLV-infected cattle [1]. Such studies using the sheep model may support the evaluation of serum bta-miR-375 as a predictive biomarker for the early diagnosis of EBL in cattle. In addition, we were unable to compare results for EBL cattle between females and males because most male calves were delivered to the calf market at an early stage on the farms that cooperated in the study. Although confirming data are unavailable, we consider it likely that serum miR-375 is elevated in male EBL cattle as in females.

In conclusion, our current results point to serum bta-miR-375 as a promising biomarker for the early diagnosis of EBL, and we recommend its use in combination with conventional biomarkers. To further assess the utility of bta-miR-375, we need to evaluate cases of sporadic bovine leukosis (SBL) not caused by BLV [1] to identify bta-miR-375–related differences between SBL and EBL. The ability to diagnose EBL early on farms before cattle show typical clinical signs will contribute to the development of methods to predict the onset of EBL.

## Supporting information

S1 Table Cattle used in this study. (XLSX)

S1 Fig Histopathology and immunohistochemistry of lymphoma tissue. Lymphoma tissue from EBL cattle was fixed in 10% neutral buffered formaldehyde, paraffine-embedded, and stained with hematoxylin and eosin (A), anti-CD3 antibody (for T cells) (B), or anti-CD20 antibody (for B cells) (C). Representative sections are shown. Bar, 20 μm.
(PPTX)

S2 Fig Correlation between bta-miR-375 and age in cattle infected with BLV but without clinical signs. A: All cattle, B: HF cattle, C: JB cattle. bta-miR-375 levels are indicated as relative quantities normalized to the synthetic spike-in control, cel-miR-39-3p. Age is indicated in months. Data were analyzed by using Spearman’s correlation coefficient test.
(PPTX)

S3 Fig Correlation between bta-miR-375 in serum and that in B cells from BLV negative (n = 2) and BLV-infected NS (n = 4) cattle and lymphoma tissue from EBL cattle (n = 4). Serum bta-miR-375 levels are indicated as relative quantities (RQ) normalized to the synthetic spike-in control, cel-miR-39-3p. Bta-miR-375 levels are indicated as relative quantities (RQ) normalized to the expression of bta-miR-16a. Data were analyzed by using Spearman’s correlation coefficient test.
(PPTX)

S1 Raw data (XLSX)

S2 Raw data (XLSX)

S3 Raw data (XLSX)

S4 Raw data (XLSX)

S5 Raw data (XLSX)

S6 Raw data (XLSX)
