# Supplementary figures and images for: Serum bta-miRNA-375 as a potential biomarker for the early diagnosis of enzootic bovine leukosis
Source: PLoS One. 2024 May 9;19(5):e0302868. doi: 10.1371/journal.pone.0302868 (PMC11081263; doi:10.1371/journal.pone.0302868)

## Slide 1
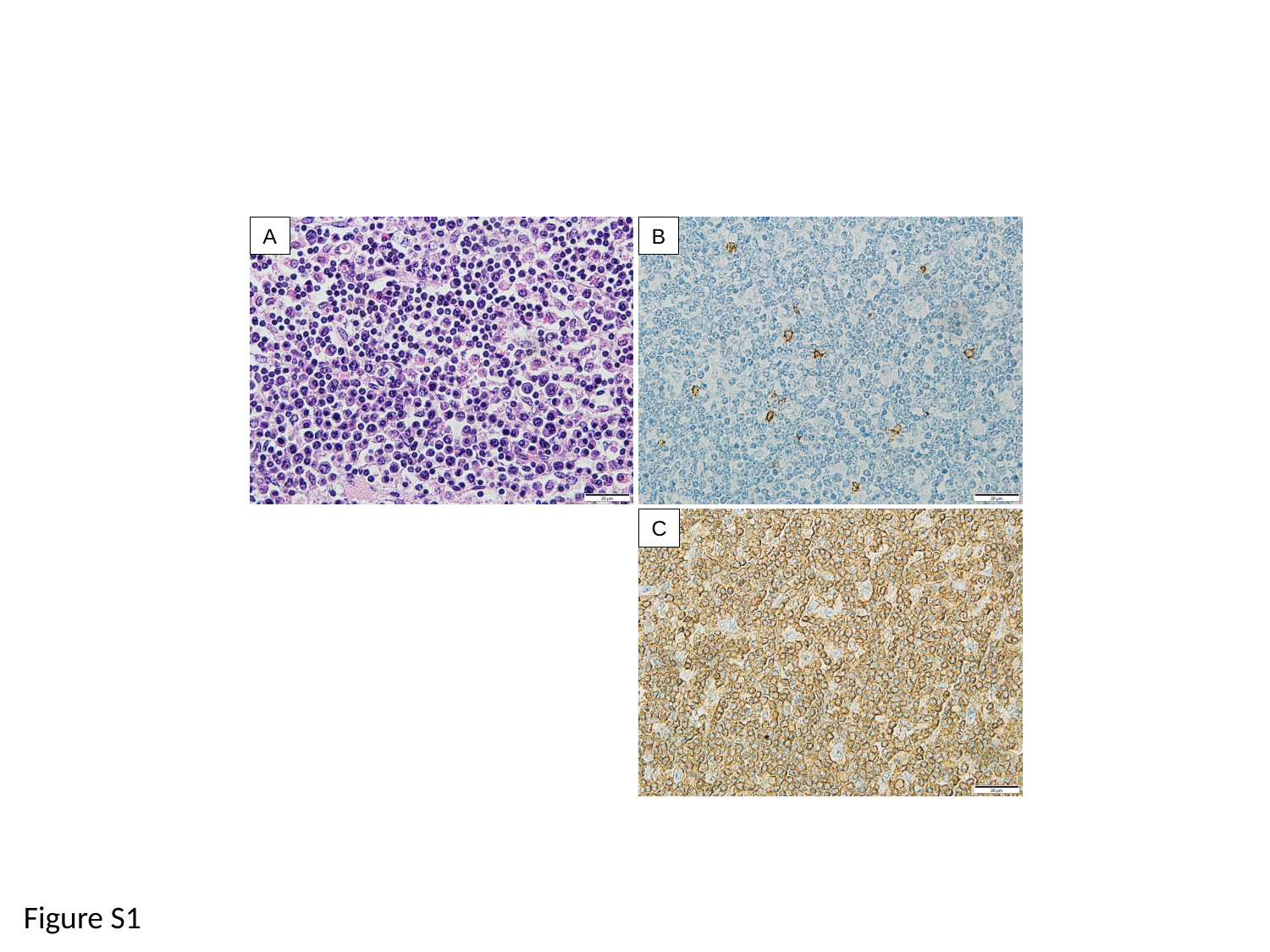

A
B
C
Figure S1

Supplement: S1 Fig — Lymphoma tissue from EBL cattle was fixed in 10% neutral buffered formaldehyde, paraffine-embedded, and stained with hematoxylin and eosin (A), anti-CD3 antibody (for T cells) (B), or anti-CD20 antibody (for B cells) (C). Representative sections are shown. Bar, 20 μm. (PPTX) [file pone.0302868.s002.pptx]

## Slide 1
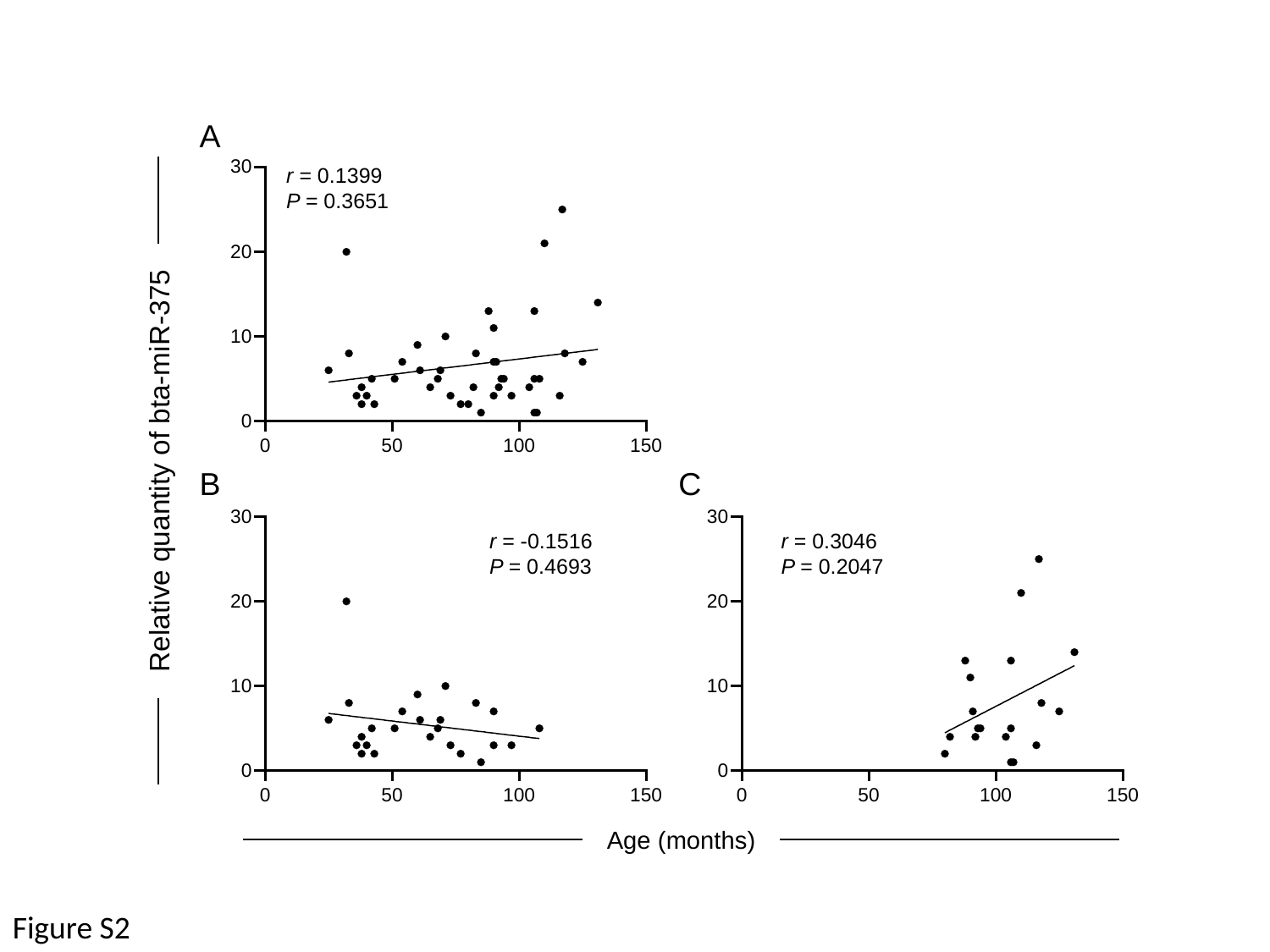

A
Relative quantity of bta-miR-375
r = 0.1399
P = 0.3651
B
C
r = -0.1516
P = 0.4693
r = 0.3046
P = 0.2047
Age (months)
Figure S2

Supplement: S2 Fig — A: All cattle, B: HF cattle, C: JB cattle. bta-miR-375 levels are indicated as relative quantities normalized to the synthetic spike-in control, cel-miR-39-3p. Age is indicated in months. Data were analyzed by using Spearman’s correlation coefficient test. (PPTX) [file pone.0302868.s003.pptx]

## Slide 1
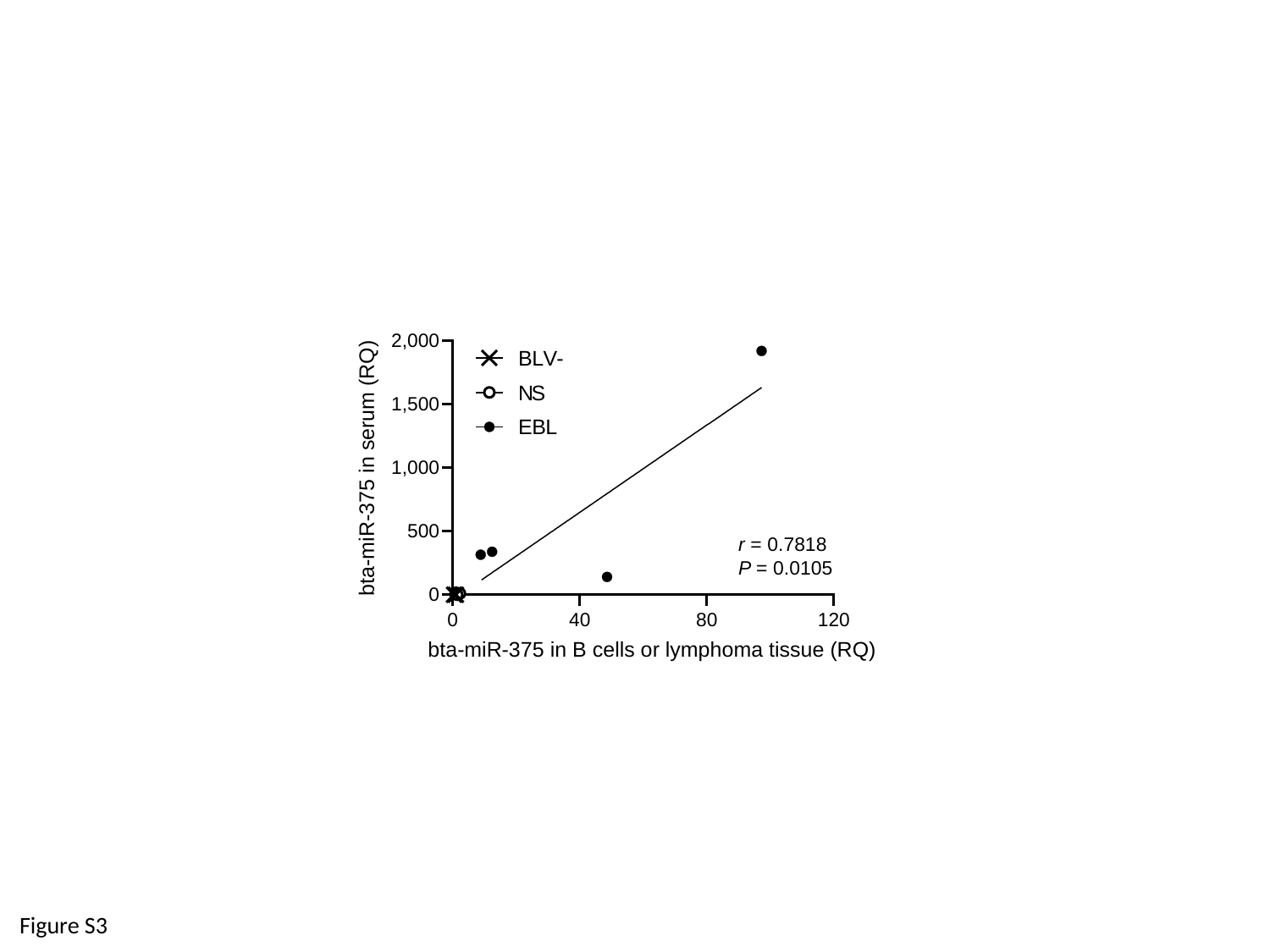

bta-miR-375 in serum (RQ)
r = 0.7818
P = 0.0105
bta-miR-375 in B cells or lymphoma tissue (RQ)
Figure S3

Supplement: S3 Fig — Serum bta-miR-375 levels are indicated as relative quantities (RQ) normalized to the synthetic spike-in control, cel-miR-39-3p. Bta-miR-375 levels are indicated as relative quantities (RQ) normalized to the expression of bta-miR-16a. Data were analyzed by using Spearman’s correlation coefficient test. (PPTX) [file pone.0302868.s004.pptx]
